# Simultaneously precise frequency transfer and time synchronization using feed-forward compensation technique via 120 km fiber link

**DOI:** 10.1038/srep18343

**Published:** 2015-12-22

**Authors:** Xing Chen, Jinlong Lu, Yifan Cui, Jian Zhang, Xing Lu, Xusheng Tian, Cheng Ci, Bo Liu, Hong Wu, Tingsong Tang, Kebin Shi, Zhigang Zhang

**Affiliations:** 1 State Key Laboratory of Advanced Optical Communication System and Networks, School of Electronics Engineering and Computer Science, Peking University, Beijing, 100871, China; 2 State Key Laboratory for Mesoscopic Physics, School of Physics, Peking University, Beijing 100871, China; 3 School of Electronic Information and Optical Engineering, Nankai University, Tianjin 300071, China; 4 Beijing Satellite Navigation Center, Beijing 100094, China

## Abstract

Precision time synchronization between two remote sites is desired in many applications such as global positioning satellite systems, long-baseline interferometry, coherent radar detection and fundamental physics constant measurements. The recently developed frequency dissemination technologies based on optical fiber link have improved the transfer instability to the level of 10^−19^/day at remote location. Therefore it is possible to keep clock oscillation at remote locations continuously corrected, or to reproduce a “virtual” clock on the remote location. However the initial alignment and the correction of 1 pps timing signal from time to time are still required, besides the highly stabilized clock frequency transfer between distant locations. Here we demonstrate a time synchronization based on an ultra-stable frequency transfer system via 120-km commercial fiber link by transferring an optical frequency comb. Both the phase noise compensation in frequency dissemination and temporal basis alignment in time synchronization were implemented by a feed-forward digital compensation (FFDC) technique. The fractional frequency instability was measured to be 6.18 × 10^−20^ at 2000 s. The timing deviation of time synchronization was measured to be 0.6 ps in 1500 s. This technique also can be applied in multi-node fiber network topology.

During the past decades, the progress in atomic/optical clocks has driven the frequency instability to 10^−15^–10^−18^/s level^1^,^2^. Yet the accuracy of transferring these frequency standards under conventional schemes based on free-space microwave propagation did not meet the requirement for comparison and distribution of such high quality frequency standard^3^,^4^,^5^,^6^. As the fiber link is more insusceptible to the ambient perturbations in comparison with free space propagation, it has attracted extensive studies on the frequency dissemination and comparison over optical fiber network^7^,^8^,^9^,^10^. There are basically three schemes adapted in the optical fiber based frequency dissemination, which have been developed in parallel for the best precision and simplicity. Those modes are: ① the RF modulated continuous wave (CW) laser^11^,^12^,^13^,^14^,^15^, ② the optical frequency standard (optical clock)^10^,^16^,^17^,^18^,^19^ and ③ the mode locked pulse trains (or a segment of frequency comb)^20^,^21^,^22^,^23^,^24^. Furthermore, there were also demonstrations of stable frequency distribution over fiber network with complex topological structure^25^.

For absolute time synchronization over distant locations, monitoring and aligning of temporal bases between two sites are indispensable in addition to frequency dissemination, which only synchronizes the “clock” oscillations. Previous work^26^,^27^ primarily focused on characterizing and controlling the timing jitter or phase variation, instead of the absolute alignment of the 1 pps (pulse per second) temporal basis and one-way comparison and feedback technique was often employed^28^. As a result, a residual time difference at the receiver end is inevitable. Recently the MPQ group has demonstrated the optical time and frequency transfer through fiber links as long as 1840 km^17^,^29^. However, the demonstrated system only targeted to establish highly precise frequency dissemination between two stations. Simultaneous time and frequency transfer over free space optical link was demonstrated by the NIST group^30^ with the use of free-space optical link recently, the system became more configurable and flexible. The ultimate performance will be limited by the atmospheric environment.

In this paper, we report a timing basis synchronization system with a feed-forward digital compensation (FFDC) scheme^31^ based precision transferred frequency over a 120 km fiber link by using mode locked laser pulses. The proposed scheme can provide a solution to construct the time-frequency transfer network, and can be extended to multi-node distribution. The measured time synchronization instability was sub-40 ps at the remote receiver end (the RMS of 12,000 data points). The timing deviation (TDEV) can be reduced to as low as < 1 ps for the integration time of 1000 s. The fractional frequency instability has been reduced to 6.18 × 10^−20^ at 2000 s.

## Results

**Figure 1:**
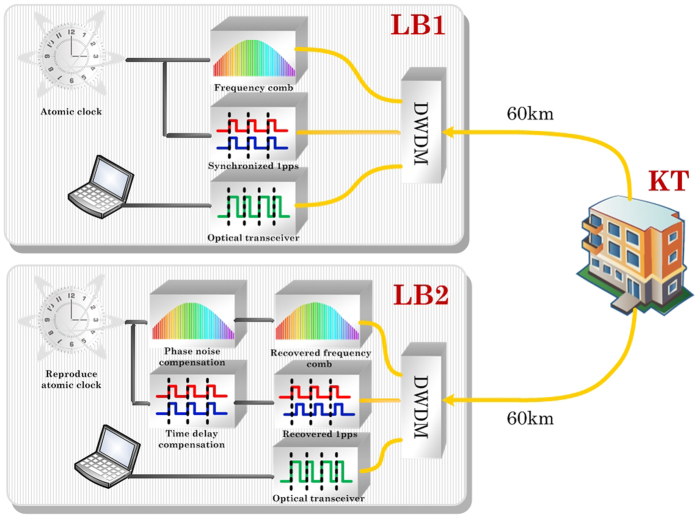
The schematic diagram of precise time synchronization and frequency transfer. At the remote end, the atomic clock can be reproduced. DWDM: dense wavelength division multiplexing.

The time synchronization of a 1 pps was built based on the precision frequency transfer system. As shown in Fig. 1, a pair of 60-km long telecommunication field fibers connects the two systems at the location of Long-Bridge town (LB) and Kintang (KT) town where a bi-directional erbium doped fiber amplifier (bi-EDFA) was placed. The total length and the loss of the fiber link measured by an OTDR (Optical Time Domain Reflectometer) were 120 km and 40.11 dB respectively. Both the sender and receiver were located at the LB station marked as LB1 and LB2, for the system evaluation. The sender LB1 and the receiver LB2 units were arranged in the same instrument rack. The whole link includes 5 bi-EDFAs at KT station and other two different stations to compensate for 40.11 dB of the fiber loss. A dense wavelength division multiplexing (DWDM) was used in the bi-directional transferring system where forward and backward signals running in six wavelength channels from the DWDM ITU-grid (channel #33 and #34 are used to transfer frequency, #30 and #31 for time synchronization, #36 and #37 for data communication respectively) without interfering each other.

### Ultra-stable frequency transfer with a segment of optical frequency comb

**Figure 2:**
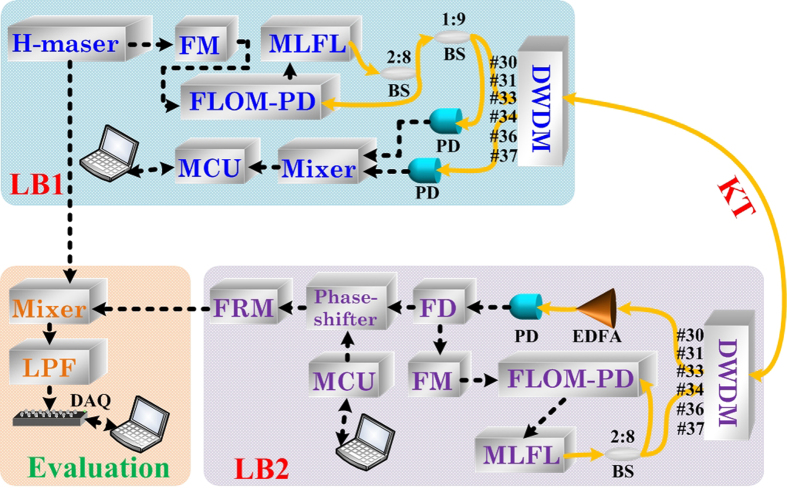
The principle of feed-forward compensation system to synchronize frequency reference. MLFL: mode locked fiber laser; FM: frequency multiplier; FLOM-PD^20^: Fiber loop optic microwave phase detector; MCU: microprocessor control unit; BS:beam splitter; PD: photo detector; EDFA: Erbium doped fiber amplifier; FD: frequency divider; DAQ: data acquisition; FRM: frequency recovery module; LPF: low pass filter.

Establishing transfer of frequency oscillation over distance is the essential basis for precise time synchronization. The schematic of the frequency dissemination system is shown in Fig. 2. Briefly, a radio frequency signal of 900 MHz was first generated by the frequency multiplier (FM) module referenced to a universal time coordinated (UTC) H-maser. The 900 MHz signal then was fed to a FLOM-PD^20^,^31^ to lock the 100 MHz repetition rate of the mode-locked fiber laser (MLFL). When successfully synchronized with H-maser, the laser pulses passed through the DWDM channel #33 with 100 GHz bandwidth and propagated to the remote end. There were about 1000 comb lines transmitted with the locked frequency spacing of 100 MHz. At remote end, the downloaded radio frequency was used to lock the other MLFL via a FLOM-PD. The newly generated laser pulses were sent back to the local site for phase noise detection in the fiber link. The feed-forward digital phase noise compensation was employed, in contrast to the conventional feedback technology. Details are discussed in the Method section. Briefly, the phase noise is digitally corrected at the remote end, rather than performed at the local end. It was carried out by the phase shifter and the frequency recovery module (FRM) as shown in the Fig. 2. The distinct feature of this technique is the instantaneous and accurate phase correction for a long fiber link. With this technique we have achieved ultra-stable frequency transmission in 120 km fiber link^31^. The high frequency stability paved the sound basis for the time synchronization.

### Two-way fiber time synchronization

**Figure 3:**
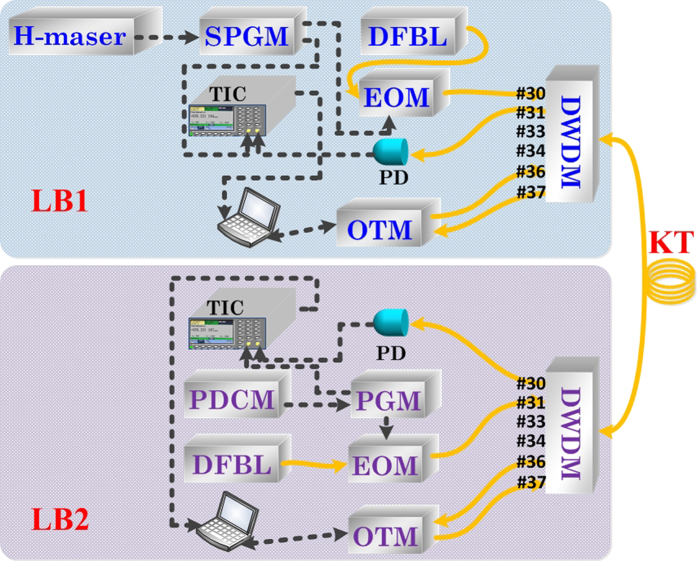
The principle of two-way fiber time transfer (TWFTT) system to synchronize the time references. TIC: time interval counter; DFBL: distributed feedback laser; EOM: electro-optic modulator; SPGM: synchronization pulse generation module; PDCM: pulse delay control module; PGM: pulse generate module; OTM: optical transceiver module.

For time synchronization, two-way fiber time transfer (TWFTT) technique was employed for the initial timing alignment. On the local site LB1 as shown in Fig. 3, the 1 pps signal generated from the UTC H-maser was used as the external trigger to the synchronization pulse generation module (SPGM) consisting of a 1 pps distributor and a pulse generator (SRS DG645). The output 1 pps signal with 100 ps rising edge, 20 μs width and 5 V level was used to drive a 10 GHz bandwidth EO modulator to modulate a CW distributed feedback laser (DFBL). The central wavelength and the linewidth of the CW laser were 1553.33 nm and 3 MHz respectively. The output power was 10 mW. The generated optical 1 pps signal was coupled to the channel #30 of the same DWDM system as the frequency dissemination used. Similarly, on the receiver side, the timing signal was also regenerated from the modulation of a DFBL whose wavelength fits the channel #31 driven by the 1 pps electrical pulses from the pulse generate module (PGM, SRS DG645) whose time base was the recovered “virtue” atomic clock and the time delay was adjustable. The channel #31 was chosen for sending back the timing signal for time interval comparison. The absolute timing difference was obtained by comparing the time intervals of the two counter-propagated 1 pps pulses by two time-interval counters (TIC, Agilent 53230 A) at both sides. The absolute timing difference Δ*T* was then feed-forward to the receiver at LB2, to reset the time delay of the 1 pps pulses generation through the VB (Visual Basic) program to ensure 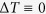 when the rising edge of the 1 pps pulses are aligned in time remotely.

### Fractional frequency instability and time synchronization measurement

**Figure 4:**
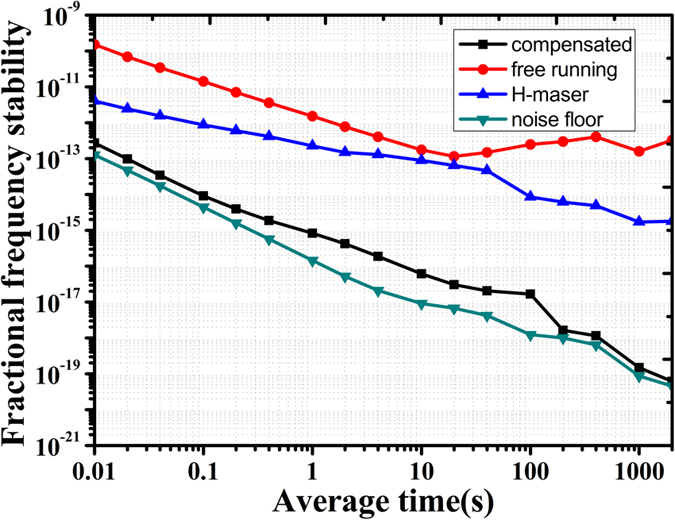
Red line: fractional frequency stability of the 120 km free running fiber link (Π data), Blue line: frequency instability of H-maser; Black: modified ADEV of fractional frequency by averaging (overlapping Λ-type) calculation; Green: the instability (modified ADEV) of system background floor (replacing fiber link with 3 m long fiber).

As shown in Fig. 2, after passing through the phase shifter and the FRM at LB2, the compensated frequency signal and the reference frequency from H-maser, was used for the dissemination stability evaluation. The frequency of 900 MHz was used. The frequency instability was processed by a data acquisition card (NI 6251) which records the output voltage of the phase comparison module consisting of frequency mixer and low pass filter (LPF) at the sampling rate of 100 Hz. Recorded output voltage from phase comparison detector was used for calculating the fractional frequency instability^3^. Figure 4 shows the fractional frequency instability of free-running and compensated at the working bandwidth of 1 Hz. The instability of the H-maser and the system noise floor is also plotted. The noise floor was measured by replacing a long haul fiber link with a 3 m long single mode fiber and the signal intensity was adjusted to the same level of the long fiber. The instability of the 120 km free running fiber link system (Π type) was measured as 1.52 × 10^−12^ at 1 s and 1.6 × 10^−13^ at 1000 s. With the digital feed-forward phase compensation, the instability (Λ type) was reduced to 8.21 × 10^−16^ at 1 s, 1.48 × 10^−19^ at 1000 s and reached 6.18 × 10^−20^ at 2000 s. The modified ADEV includes an additional phase averaging process, and can separate the white phase noise from flicker phase modulation noise. The curve-fitting shows that the frequency stability is proportional to the 1/τ, indicating the residual noise is white phase noise dominated^32^,^33^.

**Figure 5:**
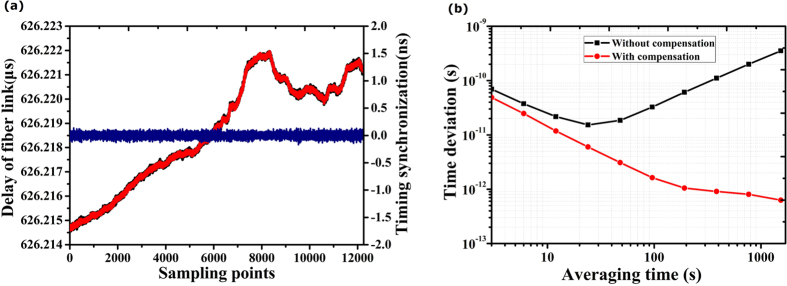
(**a**) The measured instability of the synchronization and stability of the time dissemination system with 12,000 sampling points at 0.333Hz sampling rate. The delay of fiber link and the time synchronization. The blue line is the accuracy of time synchronization. The red and black curves are the 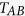 and the 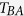 respectively. They are almost overlapped, showing the excellent synchronization; (**b**) Time deviation (TDEV) measures the instability of the timing transfer for with and without frequency stabilization.

The time synchronization measurements were performed by directing logging the time intervals acquired by the two TICs at both sites. As shown in Fig. 5(a), the delay variation of 120 km fiber link at free-running is about 7.5 ns during the time of 36,000 s (sampling points of 12,000 with the sampling rate of 0.333 Hz). By taking difference between the read-outs from two TICs, the time synchronization can be obtained as shown in the blue line in Fig. 5(a). The RMS variation of time synchronization is sub-40 ps (12,000 points averaging). This timing jitter improvement can also be evaluated by the time deviation (TDEV) as shown in Fig. 5(b). The calculated TDEV is 1.6 ps in 100 s and fells to 0.6 ps in 1500 s.

## Discussions

We have demonstrated time synchronization with ultra-stable frequency transfer using mode locked pulse train as the RF carrier over 120-km telecommunication fiber link. The fractional frequency instability was reduced to 1.48 × 10^−19^ at 1000 s, the lowest among the frequency transfer systems reported for the similar fiber length and the comparison is shown in Table 1. With the stabilized frequency as the clock, the timing was also stabilized. The RMS timing instability was < 40 ps and the TDEV of the 1 pps was as low as <1 ps at 1000 s. The TDEV measurement shows the equal level as in ref. 28 in long evaluation time.

Figure 5 shows that the instability in timing goes down to 1 ps over 1000 seconds, which means that the relative precision is 10^−15^ in timing. However, the frequency instability measured in one second average time here is the about three orders lower. The discrepancy between the timing and the frequency instability can be attributed to the timing generation and the measurement technique in our experiment. In the synchronization system, the timing pulse should normally be one of the mode locked pulses. However, the delay time of the mode locked pulses are not able to adjust instantaneously in a larger scale. Therefore we chose to make the 1 pps optical timing pulses on both sites generate by an electro-optical modulator modulated CW laser. The modulator was driven by a pulse generator (SRS DG645) which is referenced to the synchronized frequency. This way determines that the 1 pps optical pulses possessed inevitably the jitters as the pulse generator has a nominal rising edge jitter of maximum 20 ps. On the other hand, the time interval was measured by the time interval counters (Agilent 53230A) at both sites and finding the timing difference. The time interval counters are in 12-bit digits and have the read-out precision of 1 pico-second with nominal counting resolution of 20 ps. Therefore the time deviation is mainly restricted by the resolution and jitter of the instruments. Similar discussion was reported previously^7^.

It is also demonstrated that phase compensated and time alignment can be performed digitally at the remote end. In contrast to the centralized compensation scheme where the local site will bear heavy instrumentation loads under an extended multiple nodes fiber network, our proposed scheme leads to the possibilities of ultra-precision and accurate global time–frequency distribution, satellite navigation and long-base line interferometry.

## Methods

### Phase noise detection with WDM scheme

In the commercial fiber link, the fibers cannot be spliced together, and all the FC interconnectors introduced surface reflection that mixes with the feedback signals and therefore lead to detection error. Accordingly, we employed the WDM to separate the forward and feedback signals. In our system, channel #33 of the DWDM was used to forward the 100 MHz repetition rate mode locked pulse train from LB1 to LB2, and channel #34 was used to return the pulse train to LB1. The wavelength conversion from channel #33 to #34 was achieved by launching a regenerated pulse train generated by another mode locked erbium doped fiber laser, whose repetition rate was locked to the forwarded pulse train via another FLOM-PD. The spectral filtering effect of the DWDM also makes the system more insusceptible to dispersion of the long haul fiber link. The phase noise between the local and the returned signals then were detected by a frequency mixer. The advantage of this WDM scheme is that the transferred and the feedbacked signals are in the same fiber link without need to splice and is more practical.

### Feed-forward digital phase stabilization

The goal of frequency transfer is to precisely reproduce a local frequency reference signal at the remote user end. The phase noise can be detected by mixing the forward and backward pulses in optical or in RF domain. In our 120 km fiber link, the fiber length variation due to the ambient fluctuation can be much larger than the compensable range of traditional fiber stretcher or delay line. Therefore a feed-forward digital phase compensation scheme was used. In detail, once the phase fluctuation was detected at the local end (LB1), an optical communication was established by using a pair of standard small form pluggable (SFP) optical transceivers at LB1 and LB2 respectively. Wavelength channel #36 and #37 in WDM ITU-grid were used as carrier wavelengths. Then the detected phase noise was sent to the remote end through the optical communication. The received information was used to control a RF phase shifter (AD9910) to stabilize the phase fluctuation^31^.

### Time synchronization

We define the time for the optical pulse traveling through the fiber from the local to the remote as 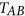, and that in reverse as 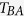, which in details are 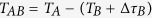 and 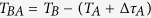 where 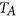 and 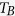 are the timing edge at which the closest 1 pps pulses are sent out at each site, 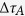 and 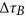 are the systematic delay including optical to electrical delay, electrical to optical conversion delay, electronics processing delay, as well as optical fiber link transmission delay at both ends. We can approximately treat 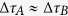 by ignoring the electrical-optical conversion time on both ends and subtracting the constant delay induced by the dispersion for different wavelength channels. In this way, the alignment error 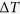 can be expressed as 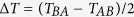 and should be set to zero at the remote site.

Similar to the frequency transfer, the time synchronization was carried out using the FFDC scheme. The alignment of the 1 pps time base was performed at the remote end (LB2). Therefore the timing difference of 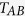 was first digitized and then sent to LB2 via data communication established upon wavelength channels of #36 and #37. After the initial alignment was made, this procedure was repeated every three seconds, to keep the timing difference 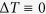, for ensuring the time synchronization all the time.

## Additional Information

**How to cite this article**: Chen, X. *et al.* Simultaneously precise frequency transfer and time synchronization using feed-forward compensation technique via 120 km fiber link. *Sci. Rep.* **5**, 18343; doi: 10.1038/srep18343 (2015).

## Figures and Tables

**Table 1:** Comparison of the time synchronization over fiber.

Organization	Carrier	Fiber length (km)	Fractional frequency stability		Time jitter (TDEV)		Year
			1 s	1000 s	100 s	1000 s	
UP13/SYRTE	Optical frequency	540	~2 × 10^−14^	~3 × 10^−17^	~7 ps	~6 ps	2013^33^
MPQ/PTB	Optical frequency	1840	2.7 × 10^−15^	—	—	—	2013^29^
NPL	Comb	86	5 × 10^−15^	~6 × 10^−17^	—	—	2011^24^
AGH Univ. Sci. & Techno.	Radio frequency	60	~6 × 10^−14^	~9 × 10^−16^	~1.6 ps	~0.7 ps	2012^28^
This work	Comb	120	8.21 × 10^−16^	1.48 × 10^−19^	1.6 ps	0.7 ps	2015
